# Antibacterial Potential Assessment of Jasmine Essential Oil Against *E. Coli*

**DOI:** 10.4103/0250-474X.41465

**Published:** 2008

**Authors:** C. C. Rath, S. Devi, S. K. Dash, R. K. Mishra

**Affiliations:** Centre for P. G. Studies. Department of Microbiology, Orissa University of Agriculture and Technology, Bhubaneswar-751 003, India

**Keywords:** Jasmine essential oil, *Jasminum sambac*L. antibacterial activity, *E. coli*, phenol coefficient

## Abstract

The antibacterial activity of Jasmine (*Jasminum sambac* L.) flower hydro steam distilled essential oil, synthetic blends and six major individual components was assessed against *Escherichia coli* (MTCC-443) strain. The activity was bactericidal. Minimum inhibitory concentration was determined by tube dilution technique, and the Minimum inhibitory concentration ranged between 1.9-31.25 μl/ml. Phenolcoefficient of the oil, synthetic blends and components varied between 0.6-1.7. The activity of the chemicals was possibly due to the inhibition of cell membrane synthesis.

In India, Jasmine (*Jasminum sambac*, sans- *mallika* ) is extensively used in manufacturing high grade aromatherapy, cheaper synthetic oil obtained by blending a few constituents are used incenses, room fresheners and soaps etc. Juices from the leaves of *J. sambac* are applied to treat ulcers, remove corns, effecting in expelling worms, regulating menstrual flow, to clean kidney waste, inflamed and blood-shot eyes. But hardly there is any report in literature regarding the antimicrobial activity of Jasmine flower essential oil. An attempt in this view is thus, undertaken to explore the potentialities of jasmine natural essential oil and its synthetic components for their efficacy against *E. coli* MTCC-443 strain.

Jasmine essential oil was extracted from flowers by hydro-steam distillation and the analysis was carried out by gas chromatography and gas chromatography-mass spectroscopy at Regional Research Laboratory (CSIR), Bhubaneswar. Synthetic oil blend was prepared mixing major constituents as per concentrations present in natural oil. Two more blends Complex-1 and Complex-2 were prepared using linalool, benzyl acetate, methyl anthranilate, and methyl salicilate as per concentrations present in natural oil and at one to one proportions respectively (Table 1). Methyl benzoate and benzyl benzoate along with other four constituents were also used in this study.

**TABLE 1:** COMPOSITION OF THREE SYNTHETIC BLENDS

Constituents	Synthetic oil (%)	Complex 1 (in ml)	Complex 2 (in ml)
Cis-3-hexanol	3.0	-	-
Cis-3-hexenylacetate	4.5	-	-
Linalool (L)	59.0	4.13	0.6
Benzyl acetate (BA)	22.5	0.797	0.6
Methyl anthranilate(MA)	1.5	0.63	0.6
Methyl salicylate(MS)	2.0	0.180	0.6
Methyl benzoate(MB)	4.5	-	-
Benzyl benzoate(BB)	3.0	-	-

Dash represents absence of respective components in the synthetic blends

*Escherichia coli* (MTCC-443) were obtained from the Institute of Microbial Technology (IMTECH), Chandigarh, India. Pure culture was maintained on Nutrient Agar slants, in our laboratory and used for the study.

Nutrient Broth (NB), Nutrient Agar (NA), Mac-Conkey (Broth), Mac-Conkey Agar (MA), Sodium Taurocholate (ST) were procured from Hi-Media, Mumbai, India, Ltd. Sodium taurocholate (1.0 %) in the media was added to facilitate the miscibility of the oil. Media without essential oil and/or components served as control in all experiments, until mentioned otherwise. Antibiotic discs such as amikacin (Ak, 30 μg), ampicillin (A, 10 μg), ciprofloxacin (Cf, 10 μg), co-trimoxazole (Co, 10 μg), erythromycin (E, 15 μg), nalidixic acid (Na, 30 μg), penicillin-G (P, 10 U), polymyxin-B (Pb-300 U), trimethoprim (Tr, 125 U), triplesulpha (3S, 300 U) were procured from Hi-Media, Mumbai, Ltd. and used for the study, in order to compare the potential of Jasmine essential oil with that of the standard antibiotics.

Screening of the natural oil, synthetic oil, synthetic blends (Complex 1 and 2) and 4 major components (linalool, benzyl acetate, methyl salicylate, methyl anthranilate and benzyl benzoate) for antibacterial efficacy was studied by disc diffusion method (DDM) following the procedure described elsewhere^1^,^2^. Minimum inhibitory concentration (MIC) of Jasmine oil, synthetic blends and components were determined by two fold tube dilution technique^3^. Further, bactericidal or bacteriostatic activity of the test samples were determined by subculturing one loopful of the culture from MIC tubes on to MA plates. No growth after the incubation period indicated bactericidal nature while, growth on subculture indicated bacteriostatic nature of the oil, synthetic blends and the components.

Furthermore, an experiment was designed to estimate the efficacy of the test samples comparing them with phenol taken as standard disinfectant as reported earlier^4^. The phenol coefficient value of the oil, synthetic blends and components was calculated using the formula, phenol coefficient = highest dilution of the test component killing *E. coli* in 10 min/highest dilution of phenol killing *E. coli*. in 10 min.

The antibiogram pattern of the strain *E. coli* MTCC-443 was determined by disc diffusion method of Bauer *et al*^5^. Natural oil, synthetic oil, blends and synthetic components were loaded at respective MIC levels on presterilized filter discs and used in the study for comparison.

The oil was extracted by hydro-steam distillation in a large scale. A yield of 0.025-0.35 by weight of flowers was recovered. Analysis of the oil by GC and GC-MS reveals the presence of cis-3-hexnol, cis-3-hexenyl acetate, linalool, benzyl acetate, methyl anthranilate, methyl salicylate, β-elemene, cis jasmone, α-franasene, γ-cadinene, cis-3-hexnyl benzoate, α-murolol, α-cadinol. Benzyl benzoate, indole as major constituents, in addition as many as 60 minor components also have been detected and identified. From the fragrance point of view the blends had very superior characteristics, though its residence time on application was too short in comparison to natural oil.

From the preliminary screening by disc diffusion method, it was observed that *E. coli* MTCC-443 strain showed a degree of susceptibility to natural jasmine oil, its synthetic blends and individual components at 2.5 μl concentration (the lowest concentration tested) per disc (Table 2). The maximum activity of the synthetic blends could be attributable to the synergistic activity of the four components (in Complex-2) when present at equal amounts in comparison to other two blends and natural oil. Synergistic effect of essential oil components against bacteria and fungi have been reported in literature^3^,^6^,^7^.

**TABLE 2:** ANTIBACTERIAL ACTIVITY OF JASMINE OIL SYNTHETIC BLENDS AND COMPONENTS BY DISC DIFFUSION METHOD

Natural oil/synthetic blends/constituents	Zone sizes (mm)		
	
	2.5 μl	5.0 μl	10.0 μl
Natural oil	7.0	8.0	9.0
Synthetic oil	17.0	18.0	22.0
Complex-1	9.0	15.0	22.0
Complex-2	17.0	22.0	30.0
Linalool	20.0	24.0	26.0
Benzyl acetate	10.0	19.0	26.0
Methyl salicylate	13.0	18.0	24.0
Methyl anthranilate	10.0	15.0	19.0
Benzyl benzoate	-	-	-

Dash represents no inhibition of the organism at these concentrations by natural oil, synthetic oil, complexes and components

The minimum inhibitory concentration (MIC) value of the test samples ranged between 1.95-31.25 μl/ml. Lowest MIC value was reported with complex-2 and the three components (BA, MS and MA) when used individually Table 3. From the nature of toxicity studies it was observed that the samples are bactericidal in nature as no growth appeared on subculture onto solid Mac-Conkey agar plates from the MIC dilution tubes. Similarly the phenol-coefficient of the test samples ranged between 0.6-1.6 and *P* ≤ 0.5 indicates the statistical significance of the phenol co-efficient values. These findings corroborates with earlier experiment of MIC determination. i. e. samples with lowest MIC values showed highest phenol co-efficient.

**TABLE 3:** MINIMUM INHIBITORY CONCENTRATION (MIC) AND PHENOL CO-EFFICIENT VALUE AGAINST *E. COLI* (MTCC-443) STRAIN

Oils/Complexes/Constituents	MIC μl/ml	Phenol-Co-efficient
Natural oil	31.25	0.6
Synthetic oil	7.8	0.9
Complex-1	15.62	0.7
Complex-2	1.95	1.6
Linalool	15.62	0.7
Benzyl acetate	1.95	1.6
Methyl salicylate	1.95	1.6
Methyl anthranilate	1.95	1.6

Minimum inhibitory concentration (MIC) and phenol coefficient was determined by tube dilution method

The antibiogram pattern of the test pathogen *E. coli* (MTCC-443) showed resistance towards 80% of the antibiotics tested Table 4. A high degree of sensitivity was reported for the synthetic oil, complexes and components, when loaded at MIC levels per discs and zone sizes were well comparable to that of amikacin and polymyxin-B. But surprisingly, linalool which represented a high minimum inhibitory concentration and low phenol coefficient, showed a sensitivity zone of 19 mm, which is well comparable to other components and synthetic blends. Since, the strain was resistant to penicillin and ampicillin, implies that the bacterial activity of Jasmine oil and its synthetic components is through some other mechanism than cell wall synthesis. Susceptibility of the strain to amikacin and polymyxin-B, further, indicates that, the possible mode of action of the oil and synthetic components may be due to the inhibition of cell membrane synthesis, specifically inhibiting the membrane proteins. Senhaji *et al*^8^, observed the antibacterial activity of essential oil from *Cinnamum zeylanicum* against *Escherichia coli* 0157:H7 is through outer membrane disintegration and increasing the permeability to ATP through cytoplasmic membrane. Similarly, Rath *et al*^9^, also reported the anti staphylococcal activity of Juniper and Lime essential oils against methicillin resistant *Staphylococcus aureus* (MRSA) through inhibition of cell membrane synthesis that corroborates with the findings observed in this investigation. The antibacterial activity of essential oils through membrane inhibition could be attributable to the hydrophobicity of essential oils, enables them to make partitions in the membrane, rendering permeability and leading to leakage of cell contents resulting in death of microbial cells^10^–^12^.

**TABLE 4:** ANTIBIOGRAM PATTERN OF *E. COLI* (MTCC-443) AGAINST GROUP SPECIFIC ANTIBIOTICS, NATURAL OIL, SYNTHETIC BLENDS AND CONSTITUENTS

Organism	Antibiotics		Oils/Blends/Synthetic Components							
		
	Sensitive to	Resistant to	O	SO	C1	C2	L	BA	MS	MA
*E. coli* (MTCC-443)	Ak(21),Pb(14)	Na,Co,E,Cf,P,Tr,A,3S	9	17	11	24	19	24	22	21

Values represented are zone sizes in mm. Oils, blends and constituents were loaded at respective MIC levels per disc. O – Natural oil; SO – Synthetic oil; C1 – Complex-1; C2 – Complex-2; L. Linalool; BA – Benzyl acetate; MS – Methyl salicylate; MA – Methyl anthranilate

In conclusion, this investigation amply proved the antibacterial activity and mechanism of action of *Jasminum sambac* natural oil and its synthetic blends against *E. coli* MTCC-443 strain.
